# Aberrant Methylation of Tumour Suppressor Gene *ADAM12 *in Chronic Lympocytic Leukemia Patients: Application of Methylation Specific-PCR Technique

**DOI:** 10.31557/APJCP.2021.22.1.85

**Published:** 2021-01

**Authors:** Amira Mohamad, Rosline Hassan, Azlan Husin, Muhammad Farid Johan, Sarina Sulong

**Affiliations:** 1 *Human Genome Centre, School of Medical Sciences, Universiti Sains Malaysia, 16150 Kubang Kerian, Kelantan, Malaysia. *; 2 *Department of Hematology, School of Medical Sciences, Universiti Sains Malaysia, 16150 Kubang Kerian, Kelantan, Malaysia. *; 3 *Department of Medicine, School of Medical Sciences, Universiti Sains Malaysia, 16150 Kubang Kerian, Kelantan, Malaysia. *

**Keywords:** ADAM12, chronic lympocytic leukemia, methylation, tumour suppressor gene

## Abstract

**Objective:**

Chronic Lymphocytic Leukemia (CLL) is a common leukemia among Caucasians but rare in Asians population. We postulated that aberrant methylation either hypermethylation or partial methylation might be one of the silencing mechanisms that inactivates the tumour suppressor genes in CLL. This study aimed to compare the methylation status of tumour suppressor gene, *ADAM12*, among CLL patients and normal individuals. We also evaluated the association between methylation of *ADAM12* and clinical and demographic characteristics of the participants.

**Methods:**

A total of 25 CLL patients and 25 normal individuals were recruited in this study. The methylation status of *ADAM12* was determined using Methylation-Specific PCR (MSP); whereas, DNA sequencing method was applied for validation of the MSP results.

**Results:**

Among CLL patients, 12 (48%) were partially methylated and 13 (52%) were unmethylated. Meanwhile, 5 (20%) and 20 (80.6%) of healthy individuals were partially methylated and unmethylated, respectively. There was a statistically significant association between the status of methylation at *ADAM12* and the presence of CLL (p=0.037).

**Conclusion:**

The aberrant methylation of *ADAM12* found in this study using MSP assay may provide new exposure to CLL that may improve the gaps involved in genetic epigenetic study in CLL.

## Introduction

A disintegrin and metalloproteinases (ADAMs) consist of more than 30 proteins of Metzincin family. They are all matrix zinc-dependent proteases enzymes, multifunctional proteins, and principally membrane-bound family of multidomain (Edwards et al., 2008; Seals and Courtneidge, 2003). In this family, there are forty gene members, and only 21 of them appear in human and is thought to function in human body (Duffy et al., 2011). ADAMs family members have various roles, including cell signalling (Edwards et al., 2008; Seals and Courtneidge, 2003), protein processing (Eichenauer et al., 2007; Hundhausen et al., 2007; Roghani et al., 1999), adhesion (Seals and Courtneidge, 2003), fertility (Edwards et al., 2008), and proteolytic shedding of receptor ectodomains (Edwards et al., 2008; Mochizuki and Okada, 2007). All these diversified multifunctions are reflected in their structures which can be subdivided into head, body, and tail. Head of the proteins consists of pro and catalytic domains. These domains mediate processing of growth factors and cytokines by ectodomain shedding and have been in charge of epidermal growth factor (EGF) and insulin-like growth factor (IGF) receptor signalling. The body of the proteins which consists of disintegrin, cysteine-rich, and EGF-like domains is in contact with the extracellular matrix and other cells through interactions with integrins and syndecans. The tail of the proteins which consists of cytoplasmic domain is in contact with the extracellular matrix and other cells through interactions with integrins and syndecans. The tail is also in interactions with signalling molecules. Furthermore, splice forms lie in some ADAMs, such as ADAM9, ADAM28, and ADAM12, shorter secreted and soluble forms have been discussed (Duffy et al., 2009; Edwards et al., 2008; Jacobsen and Wewer, 2009; Mochizuki and Okada, 2007; Murphy, 2008; Ohtsu et al., 2006). 

ADAM12 is a proteolytic member of the ADAMs family that, as a result of alternate splicing, encodes for two different forms. Membrane-bound form (ADAM12-L) is the first form, which has different types of domains, including pro-domain, metalloproteinase domain, disintegrin domain, cysteine-rich domain, EGF-like domain, transmembrane region, and cytoplasmic tail . Another form is secreted one (ADAM12-S), which has all the aforementioned domains except the transmembrane and cytoplasmic domains (Gilpin et al., 1998; Kurisaki et al., 1998; Yagami-Hiromasa et al., 1995). The *ADAM12 *gene is located on chromosome 10q26 in human gene and reported as meltrin α by (Yagami-Hiromasa et al., 1995), a protein involved in myoblast fusion.* ADAM12 *is up-regulated in bladder (Fröhlich et al., 2006), stomach (Carl-McGrath et al., 2005), and liver cancers (Le Pabic et al., 2003). Through induction of stromal cell apoptosis, ADAM12 protein can stimulate the progression of breast tumour.

ADAM12 is highly expressed in tissues characterised by exaggerate growth, such as human placenta and tumours (Jacobsen and Wewer, 2009; Kveiborg et al., 2008; Wewer et al., 2006). Its expression is higher in malignant tumor cells, but lower in most normal adult tissues (Iba et al., 2000). It is also associated with the spreading and progression of human cancers (Fröhlich et al., 2006). In three different studies, it was shown that *ADAM12* controlled tumour progression in gene modified mice models (Kveiborg et al., 2005; Peduto et al., 2006; Sørensen et al., 2008). *ADAM12* also has been recognized as one of the candidate cancer genes in a comprehensive mutational analysis on human breast cancer. In another study on 122 genes, it was found that this gene was mutated with high frequencies in breast cancers, there was only one *ADAM* gene, namely *ADAM12* and the rest only 14 genes had a higher cancer mutation prevalence score than* ADAM12*. In the aforementioned study, it was suggested that *ADAM12* had a critical function in breast cancer progression considering a high mutation frequency together with a strongly up-regulated expression of* ADAM12* (Sjöblom et al., 2006). Breast cancer cases are commonly associated with raised levels of ADAM9, ADAM12, ADAM15, ADAM17, and ADAM28 (Kuefer et al., 2006; Lendeckel et al., 2005; O’Shea et al., 2003). The current study was attempted to first compare the status of hypermethylation in CLL patients and normal individuals and second to discover any associations between the hypermethylation status and demographic data. 

## Materials and Methods

*Sample size estimation*

Sample size was estimated using sample size calculation software (PS software). The sample size for this research was calculated for a dichotomous independent case-control study with a ratio of 1:1 CLL patients and normal controls. Expected frequency of exposure was 89% and 14.3% in control group and CLL patients group, respectively. In control group, 89% exposure was based on the results of a hypermethylation study on blood mononuclear cells showing that 89% of the controls had hypermethylation at *p16INK4a *(Deligezer et al., 2006). In CLL patients group,14.3% exposure was based on the results of hypermethylation study on CLL patients in Hong Kong (Chim et al., 2006). The sample size required for the study controls to be able to reject the null hypothesis was 8 CLL patients and 8 normal. Our null hypothesis was that the exposure rates for CLL patients and controls were equal with probability (power) 95%. However, we considered CLL patients and controls. Therefore, the total number of participants was 50 in the current study.

*Patient selection and sample collection*

In this study, 25 peripheral blood samples were obtained from patients diagnosed with CLL at Universiti Sains Malaysia Hospital (Kubang Kerian, Malaysia) and Ampang Hospital (Selangor, Malaysia). In addition, twenty five peripheral blood samples were obtained from healthy donors without family history of cancer. All samples were in compliance with consent forms. 

*DNA extraction*

High-molecular weight genomic DNA was isolated from peripheral blood samples according to the manufacturer’s specifications of QIAamp^®^ DNA Blood Mini kit (QIAGEN, Hilden,Germany). Extracted DNA had concentration up to 2000 ng/µl and purity among 1.8 – 2.0. The genomic DNA isolated from peripheral blood of both groups based on standard protocols.

*Bisulfite treatment*

Bisulfite treatment of DNA is a complete deamination process of unmethylated cytosine, but not methylated cytosine to uracil . All original cytosines will be displayed as thymines; meanwhile, methylated cytosines will be remained as cytosines in the final sequence pattern. This treatment was carried out using commercially available kit, CpGenome Modification kit (Chemicon Europe Limited., UK), and according to the manufacturer’s instructions. Briefly, 250 ng of genomic DNA was incubated in mild heated water bath at an alkaline pH. At this time, the DNA bases were first exposed to its single stranded form through denaturing process. After that, incubation was done for 16 hours which led to sulfonation and hydrolytic deamination processes, producing uracil sulfonate intermediate. The resultant DNA was bound to a micro-particulate carrier in the presence of another salt provided in the kit. The conversion was completed after desulfonation and repeated desalting. The DNA was finally eluted from the carrier by heating in 25 μl elution buffer. 

*Methylation–Specific PCR (MSP)*

Specific MSP primers for *ADAM12* were designed using MethPrimer software. Details on the primers’ sequences are shown in Table 1. they were purchased from First Base Technologies Sdn Bhd. MSP analysis was performed in a total volume of 10 µl reaction mixture, which consisted of 1 X PCR Buffer, 1.5 mM MgCl_2_, 0.2 mM dNTP, 0.5 u/µL AmpliTaq Gold DNA polymerase (Applied Biosystems, US.), 0.2 µM forward and reverse primers, approximately 50 ng of genomic bisulfite treated DNA template, and distilled deionised water (ddH_2_O). MSP amplification of all genes was done and optimised using the Touchdown PCR protocol and GeneAmp^®^ PCR System 9700 (Applied Biosystems, US) machine under the following cycling conditions: 10 minutes initial denaturation (95°C), 14 cycles of 20 seconds denaturation (94°C), 1 minute annealing (variable temperature), and 1 minute elongation (72°C). Amplification was run for further 32 cycles of 20 seconds denaturation (94°C), 1 minute annealing (57°C), 1 minute elongation (72°C), and final elongation at 72°C for 5 minutes and storage at 10°C. Four µl of PCR products were loaded into 3% agarose gel, stained with Ultra Power DNA Stain (BioTeke Corporation, Beijing, China), electrophoresed and visualized under UV light, and its picture was captured by a UV Transilluminator (Wealtec Corp, Taiwan). 

*DNA sequencing*

Amplicon obtained from every MSP was considered as critical sample due to very high concentration and volume are needed prior to the purification and DNA sequencing. 

The PCR products were sent for DNA sequencing service (First BASE Laboratories Sdn Bhd, Malaysia) to validate the qualitative results observed from MSP assay. 

*Statistical analysis*

The statistical analysis was done to discover any associations between patients’ demographic data and clinicopathologic characteristics. The data were analysed using SPSS 21 and running Fisher’s Exact, Pearson Chi-square for categorical variables, and independent T-tests for continuous variables. Patients’ demographic data included in this study were age, gender, and race and patients’ clinicopathological data involved clinical features and current treatments. 

## Results

*Demographic characteristics in CLL patients and controls*

The age of CLL patients and controls ranged between 52 to 78 years and 23 to 42 years, respectively.The mean ±SD of age for CLL patients and controls were 65.8 ± 7.8 years and 27.9 ± 4.3 years, respectively. The ratio of male to female among CLL patients was 2.1:1. Therefore, predominant among male was encountered . For clinical data, two categories were assessed, namely clinical features and current treatment. In this regard, characteristics such as lymphadenopathy, hepatomegaly, and splenomegaly were reviewed in CLL patients. Patients undergoing chlorambucil or prednisolone or both treatments were classified under active treatment. The distribution of CLL patients based on clinical features and current treatment is summarised in Table 1. 

*DNA concentration and genomic integrity*

The genomic DNA samples from CLL patients, controls, and cell lines demonstrated a competitive concentration ranging between 20 and 1,400 ng/µL. The purity of the genomic DNA samples were between 1.7 and 2.0 (A260/A280). The genomic DNA samples were also evaluated for their integrity by performing standard gel analysis using 1% agarose gel.

*Determination of hypermethylation status of ADAM12*

**Figure 1:**
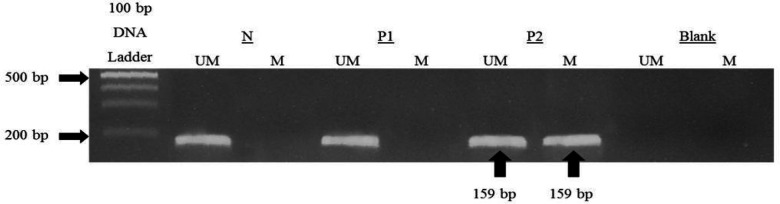
Determining the Methylation Status of *ADAM12* Using MSP Analysis. Primer sets used for amplification are designated as unmethylated (U) and methylated (M). Blank, reagent blank; Sample P, patient’s DNA; Sample N, normal DNA control. One normal sample and one patient sample showed unmethylated at *ADAM12*; whereas, another patient’s sample showed partial-methylated at *ADAM1*

All 50 bisulfite treated DNA samples were screened for the hypermethylation status using MSP analysis. For *ADAM12*, the size of the amplified amplicon was 159 bp for both unmethylated (UM) and methylated (M) *ADAM12.* If any samples showed both unmethylated and methylated bands, the sample was partially methylated (Dastjerdi et al., 2013). Blank is PCR solutions with ddH20 as the replacement of sample. Each batch of MSP analysis was repeated in duplicate. Representative results on MSP analysis of the CLL patients and controls for *ADAM12* are shown in Figure 1. 

*DNA sequencing analysis*

**Figure 2:**
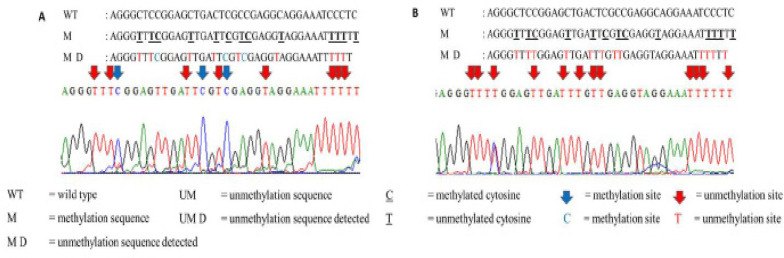
Sequencing Result of Targeted Region of Methylated *ADAM12* in Partially Methylated CLL Sample. A) At the methylation sequence detected (MD), thymine (T) marks the presence of unmethylated cytosine (T); whereas, cytosine (C) marks the presence of methylated cytosine (C). At the MD, T appears at unmethylation sites, G appears at the methylation sites, thereby confirming that the patient had partially methylated at ADAM12. (B) Electropherogram picture showed successful formation of thymines and cytosines as a result of complete conversion of unmethylated and methylated cytosines, respectively

**Figure 3.**
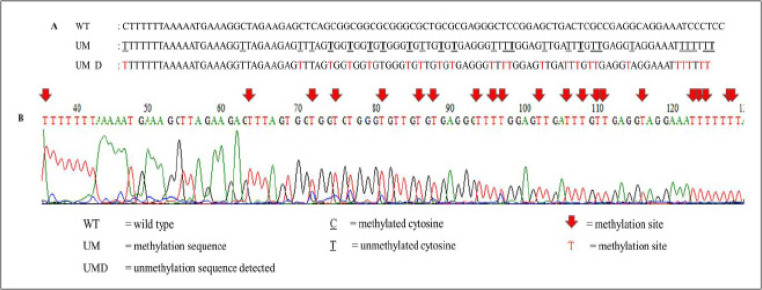
Sequencing Result of Targeted Region of Unmethylated of *ADAM12 *in Partially Methylated Patient’s Sample. A) Comparison of unmethylated wild type sequence of ADAM12 with methylated ADAM12 sequence detected in a patient sample. At the unmethylation sequence detected (UMD), thymine (T) marks the presence of unmethylated cytosine (T). B) Electropherogram picture shows successful formation of thymines however, there are some sites that show mixture of cytosine and thymine

Patient samples of MSP analysis that gave the most prominent band during gel electrophoresis were subjected to DNA sequencing. The purpose of this DNA sequencing was to validate the methylation status detected by MSP assay. Sequencing results for partially methylated and unmethylated *ADAM12* are shown in Figure 2 and Figure 3, respectively.

*Determining the association between hypermethylation status and demographic and clinical characteristics in CLL patients*

The results on association between the demographic data (age, gender and race) and clinical characteristics (clinical features and current treatment) and hypermethylation status of CLL patients are displayed in Table 2. One CLL patient did not have sufficient clinical data, so he was excluded from the study. 

**Table 1:** *ADAM12* Hypermethylation in CLL Patients and Control Group (N=50)

Status of hypermethylation at *ADAM12*	Case n=25	Control n=25	p-value
Unmethylated, n (%)	13 (52.0)	20 (80.6)	-
Partially methylated, n (%)	12 (48.0)	5 (20.0)	0.037*

*Pearson-Chi Square test, p<0.05 = statistically significant

**Table 2:** Associated Factors of Demographic and Clinical Characteristics in CLL Ppatients *ADAM12* Methylation Status

Variable	*ADAM12*		p-value^a^
	Unmethylated	Partially methylated	
	n (%)	n (%)	
Gender, n=25			
Female	5 (38.5)	3 (25.0)	0.673^a^
Male	8 (61.5)	9 (75.0)	
Race, n=25			
Malay	7 (53.8)	8 (66.7)	0.688^a^
Non-Malay	6 (46.2)	4 (33.3)	
Clinical features, n=24			
Lymph node			
Yes	8 (66.7)	10 (83.3)	0.640^a^
No	4 (33.3)	2 (16.7)	
Splenomegaly			
Yes	4 (33.3)	8 (66.7)	0.102^b^
No	8 (66.7)	4 (33.3)	
Hepatomegaly			
Yes	5 (41.7)	8 (66.7)	0.219^b^
No	7 (58.3)	4 (33.3)	
Current treatment, n=24			
Yes	7 (53.8)	6 (50.0)	0.848^b^
No	6 (46.2)	6 (50.0)	

## Discussion

A significant association between hypermethylation status of *ADAM12* and CLL was detected in this study (p=0.037). In a previous microarray study on CLL disease, Rahmatpanah et al., (2009) found that ADAM12 was methylated in CD38highpatients and high-risk CLL patients showed 31-92% of CD38 expression. Interestingly, they also discovered methylation of ADAM12 in a CLL patient’s cell line. This cell line was initially induced by cytokines and subsequently infected with Epstein-Barr virus. Association between CD38 expression and aggressive clinical behaviour of CLL was confirmed in two other studies (Ibrahim et al., 2001; Durig et al., 2002). It is commonly accepted that CD38+ patients show poor prognosis, therefore, they usually have a shorter progression-free interval, need earlier and recurrent treatments, and eventually die sooner (Damle et al., 1999). In this study, it was revealed that methylation of *ADAM12* was associated with aggressive progression of CLL and it can be contributed to the advance symptoms of CLL. ADAM12 has a variety of functions in normal cells, such as multidomain and signaling properties. However, it is excessively expressed in uncontrolled growing cells, such as breast cancer (Sjöblom et al., 2006) and other tumours. Despite these findings, studies on *ADAM12* in haematological disease are scarce when compared with non-haematological diseases, which makes the role of *ADAM12* in CLL patients remains ambiguous. However, the association of ADAM12 methylation with CLL can be supported by the fact that CLL progression is slower than other cancers, suggesting methylation of *ADAM12* may contribute to the speed of disease progression. 

Among the non-haematological findings on ADAM12 are a table of expression of a disintegrin and metalloproteinases (ADAMs) in human cancers and their possible functions. Therefore, it is suggested that ADAM12 functions as a sheddase, adhesion molecule, and ECM-degrading proteinase, and is involved in cancer progression (Mochizuki and Okada, 2007). ADAM12 is expressed in high amounts in excessively growing cells, including human placenta and tumours; whereas, the opposite happens in normal tissues (Nariţa et al., 2010). Therefore, it is associated with the metastasis of human cancers (Frohlich et al., 2006). It was proven that *ADAM12* regulated tumour progression in gene-modified mice models (Kveiborg et al., 2005; Peduto et al., 2006). A combination of high mutation-frequency and a strongly up-regulated expression of *ADAM12* in breast cancer suggest that this gene may enhance progression of breast cancer (Sjöblom et al., 2006). ADAM12 in breast cancer was among the first of ADAMs shown to have diagnostic potential. Roy et al., (2004) found that urinary levels of ADAM12 were clearly higher in breast cancer patients compared to healthy groups and the proportion of breast cancer patients having high urinary level of ADAM12 was clearly greater than the healthy groups. Follow-up studies reported the same result in patients with putative premalignant lesions of invasive breast cancer, such as atypical hyperplasia and lobular carcinoma in situ and control groups (Pories et al., 2008). Apart from that, urinary level of ADAM12 was also found to be elevated in patients with bladder cancer (Frohlich et al., 2006). 

Bisulfite-based DNA methylation analysis has higher quantitative accuracy, detection sensitivity, and efficiency. It has a wide spectrum for sample analysis when compared with restriction enzymes-based assays (Li and Tollefsbol, 2011a). An MSP analysis is a simple method without using costly sequencing reagents and exposure to radioactivity. It does not involve many sub-techniques and requires shorter time to be completed in order to obtain the actual hypermethylation status of samples. Another reliable and sensitive assay for hypermethylation detection is methylation sensitive-high resolution melting (MS-HRM) (Dhingra et al., 2014). In the MS-HRM analysis, samples are bisulfite treated and PCR amplified before they are subjected to MS-HRM assay. MS-HRM compares melting profiles of unknown PCR products to known products which are amplified from both unmethylated and methylated DNAs. Despite these advantages, this study did not utilise MS-HRM as MSP also has the advantages of being rapid and sufficiently sensitive. Besides, it is an economical technique. MSP also has the benefit of being highly sensitive due to its ability to detect single methylated allele among more than 1,000 unmethylated alleles (Dhingra et al., 2014; Herman et al., 1996). The fragments selected for MSP amplification are intentionally kept small so as to examine patterns of methylation in limited region and to conduct investigation where amplification of larger fragments is not possible. It requires the regular equipments that can be found easily in any laboratory, which makes the MSP protocol less complex when compared with MS-HRM that requires HRM machine.

Compared to Southern analysis, MSP is also significantly more sensitive. It can detect low numbers of methylated alleles from reduced quantity of DNA samples, such as the paraffin-embedded materials. This type of sample could not be analysed previously by Southern analysis (Herman et al., 1996). MSP permits analysis of all CpG sites within a whole genome, not just those within short sequences identified by methylation-sensitive restriction enzymes, allowing the fine mapping of methylation patterns throughout CpG-rich regions. MSP also can eliminate the common false positive results due to inherent partial digestion of PCR products by methylation-sensitive restriction(Herman and Baylin, 2001). An extra advantage of MSP is its immediate determination of unmethylated and methylated products in a single sample that can be used to verify the integrity of DNA as a template for PCR, thus permitting a semi-quantitative assessment of allele types that are close to the quantitation determined by Southern analysis (Herman et al., 1996). MSP also exhibits a higher sensitivity (Galm and Herman, 2005) when compared with mutation and microsatellite analysis for the detection of tumour cells in one thousand normal cells (Goessl et al., 2002). Other advantage of MSP is that it can determine DNA methylation pattern of double stranded DNA since the bisulfite treated DNAs are no longer self-complementary and the amplification of products can be measured individually (Li and Tollefsbol, 2011b). 

In summary, in this study, it was found that *ADAM12 *was one of the targets for hypermethylation in CLL. Future studies investigating the epigenetic marker in more heterogenous sample size are warranted to determine whether epigenetic dysregulation occurs in the CLL patients of Asians population or not. In conclusion, this study adds to the abundance findings of CLL and also the methylated genes specific for it. It was also able to fill many literature gaps in the genes studied for CLL. 
